# Regional co-location pattern scoping on a street network considering distance decay effects of spatial interaction

**DOI:** 10.1371/journal.pone.0181959

**Published:** 2017-08-01

**Authors:** Wenhao Yu

**Affiliations:** 1 Faculty of Information Engineering, China University of Geosciences, Wuhan, China; 2 National Engineering Research Center for Geographic Information System, Wuhan, China; Peking University, CHINA

## Abstract

Regional co-location scoping intends to identify local regions where spatial features of interest are frequently located together. Most of the previous researches in this domain are conducted on a global scale and they assume that spatial objects are embedded in a 2-D space, but the movement in urban space is actually constrained by the street network. In this paper we refine the scope of co-location patterns to 1-D paths consisting of nodes and segments. Furthermore, since the relations between spatial events are usually inversely proportional to their separation distance, the proposed method introduces the “Distance Decay Effects” to improve the result. Specifically, our approach first subdivides the street edges into continuous small linear segments. Then a value representing the local distribution intensity of events is estimated for each linear segment using the distance-decay function. Each kind of geographic feature can lead to a tessellated network with density attribute, and the generated multiple networks for the pattern of interest will be finally combined into a composite network by calculating the co-location prevalence measure values, which are based on the density variation between different features. Our experiments verify that the proposed approach is effective in urban analysis.

## 1. Introduction

With the rapid development and extensive application of ubiquitous network technology, huge collections of geospatial data become available. There is an increasing demand for the incorporation of data mining techniques into the knowledge discovery from large spatial databases. The major objective of spatial data mining is to automatically discover interesting, potentially useful, and previously unknown patterns from large amounts of geo-referenced data [1]. This process is usually realized via spatial co-location/correlation pattern mining [1–3].

Spatial co-location pattern is a subset of spatial features whose events are usually located in close spatial proximity. Finding such a pattern is one of the most important techniques for understanding geographically global relationships in spatial data sets. It should be noted that many associations in geographic context are only regional or local, rather than global due to spatial heterogeneity [3–5]. The global measurements probably lead to the loss of association information hidden at a regional scale [6]. For example, based on regional statistics, esophageal cancers is associated with water pollution in a county, while such relationship may be diluted at a larger spatial extent (e.g. state). Therefore, many domain applications have been developed in the literature focusing on the relationships at a local scale rather than a global scale [4, 7].

Based on the local variation of dependency, regional co-location pattern scoping aims to identify regions in which the spatial features under investigation are strongly co-located. The application domains of this technique are wide, including risk analysis, business mangement, LBS (location-based services) query processing, environmental studies, etc. For example, to answer LBS queries like “*Which region could I drive to for filling my car up while having a coffee nearby*?”, service providers would be interested in finding the “*hot spot*’ regions where gas station and coffee shop are most likely to be found in proximity. In addition, detecting the regions in which urban facilities and street crime have a strong association relationship would also help the public security department allocate police presence more effectively. In environmental protection, experts are devoted to identifying the risk regions related with heavy metal pollution in the water supply [4].

Although both global co-location mining and regional co-location scoping have been widely studied and applied, most of them compute neighborhood using Euclidean distance [4,8–10]. In some circumstances, however, it is necessary to scope co-locations using a different distance metric, such as network path distance, because many human activities are constrained only to the network subset of the planar space [11, 12]. For example, to satisfy the information needs of location-sensitive advertisements, mobile service providers often need to identify paths in which the co-locations of different facilities are prevalent enough [13]. These paths are spatially irregular and they may vary at multiple distance scales.

Therefore, it is necessary to develop new methods to identify 1-D paths in which different types of objects or events frequently occur together. However, the existing methods have difficulties in dealing with such issue. The major challenges to identify local paths for a pattern of interest include the following two aspects.

**Fig 1:**
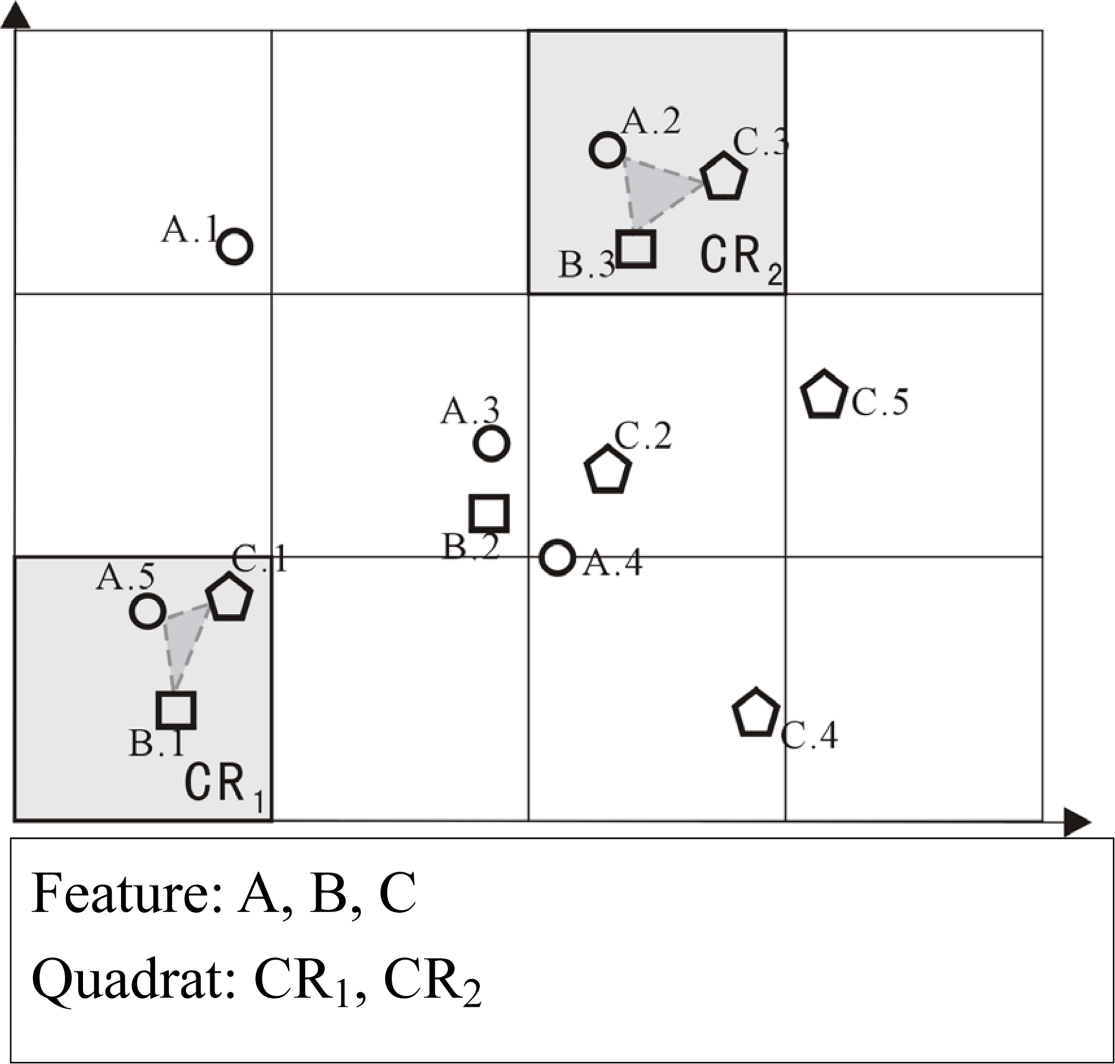
Illustration of identifying regional co-location instances and valid regions (or cells) by using traditional method.

1. *How to partition the study region into sub-regions (or cells) and compute the neighborhood envolving network-constrained features*?
  Existing methods for regional co-location scoping need to first divide the study region into equal-sized and homogeneous cells (i.e. 2-D grid), and then calculate an interestingness value for each cell by considering objects inside as a co-location intstance [3, 4]. A cell with high interestingnesss value is said to be valid with respect to the co-location pattern under investigation. However, such methods have some disadvantages: (1) the neighborhood is computed based on Euclidean distance and the result is on the whole region in which the network is embeded, regardless of the constaint of street network; (2) some of the co-location instances across the boundaries of the chosen cell are lost. For example, by using the traditional algorithm [4] on the dataset of Fig 1, events A.3, B.2 and C.2 are not considered as a co-location instance as they are not located in the same cell.
2. *How to define the co-location interestingness on each cell*?
  Previous measures (e.g., *support* and *participation index*) for co-location interestingness evaluation are based on counting, which do not take the “Distance Decay Effects” of spatial interaction into account. In other words, both types of co-location instances (having closer objects and farther objects) are treated as the same. In the geospatial domain, “Distance Decay Effect” which satisfies *first law of geography* of Tobler [14] can be presented as the strength of spatial correlation decays with distance between events. The conventional counting-based measurements cannot model and analyze such relations within a fixed boundary of neighborhood distance.

In view of these issues, this article proposes a novel approach by integrating the techniques of network analysis and distance decay function. In the approach, the valid regions of patterns of interest are constrained to continuous small linear segments, and the co-location interestingness of each place is assessed using a weighted prevalence measure, which takes into account the geographic context and spatial relationships across tessellated cells.

The organization of this paper is as follows: Section 2 reviews the related literature. Section 3 presents the improvement of co-location scoping which involves the issues about neighborhood determination and “Distance Decay Effect”. In Section 4, we demonstrate the effectiveness of our approach through a case study with real-world street network and facility POIs data. Section 5 concludes the paper and outlines further research directions.

## 2. Related work

Relevant researches can be discussed from two perspectives: *extenison from global co-location mining to regional co-location scoping* and *extension from planar space to network space*. The detail about both of the topics is as following:

### 2.1 Extension from global co-location mining to regional co-location scoping

In the last two decades, spatial co-location pattern mining has been widely studied in geo-spatial domain, aiming at measuring spatial dependence between geographic features by using spatial proximity concepts [1, 10, 15, 16, 17]. Shekhar and Huang [10] bring a systematic way to mine co-location patterns in spatial data set relying on the *participation index* measure. Given a co-location pattern P = {*f*_*1*_, *f*_*2*_,*…*, *f*_*k*_} containing a set of feature types *f*_*1*_, *f*_*2*_,*…*, *f*_*k*_, the *participation index* PI(P) is defined as min_fi∈P_{PR(P, *f*_*i*_)}, where *participation ratio* measure PR(P, *f*_*i*_) is $\mathrm{PR}(P,\;f_{i})=\frac{\mathrm{Number}\;\mathrm{of}\;\mathrm{distinct}\;\mathrm{objects}\;\mathrm{of}\;f_{i}\;\mathrm{in}\;\mathrm{instances}\;\mathrm{of}\;P}{\mathrm{Number}\;\mathrm{of}\;\mathrm{objects}\;\mathrm{of}\;f_{i}}$. Under the framework of PI measure, different variations of *Apriori* algorithm were proposed to improve the performance of instance connecting process [17–19]. Recently, Huang, Pei and Xiong [20] adjusted the interest measure to support the co-location mining evolving rare features, e.g., a rare disease. Flouvat et al. [21] integrated expert constraints inside transaction-based mining process for finding more accurate information.

However, it should be noticed that all the frameworks mentioned above are designed for specific types of global co-location patterns mining, in which valid scope of patterns is the whole study region. Our approach, on the other hand, focuses on the regional co-locations, in which valid scope of patterns is restricted to a local region. In the fields of spatial statistics and spatial data mining, regional co-location scoping attracts an increasing attention. For example, Guo et al. [22] extended classic K function to measure the local association of two variables (e.g., temples and villages). In the works of Ding et al. [3, 4], the whole area is firstly divided into a fixed 2-D grid, then the output for a co-location is formed by a set of square cells, in which the probability of occurrence of the co-location maximizes a specified fitness function. However, these works analyze the distributions of events within different cells separately, and it may lead to the loss of neighborhood (or instances) across the boundaries of cells (see Fig 1).

### 2.2 Extension from planar space to network space

Neighborhood definition is another important issue for mining spatial patterns [23]. In spatial co-location mining, different neighborhoods were proposed to meet different kinds of application requirements, including fixed-distance neighborhood [10, 24], space partition [18], buffer zone [25], topological relationship [26], k-nearest neighbors [27], and Delaunay diagram [28]. However, most of these approaches assume uniform distributions of events on a 2-D plane. The relevant measures may become inappropriate in applications of mobile services and street infrastructure planning due to the factor that the movement in urban areas is usually constrained by the street network. In the fields of geo-computation and spatial statistics, the issue of network space versus Euclidean space has attracted attention of many scholars. For example, Okabe et al. [29] and Ai et al. [30] proposed to use network distance rather than Euclidean distance to construct Voronoi diagram. Yamada and Thill [7, 31] developed a constrained *K*-function based on network distance to analyze the cluster characteristics of traffic accident data. Shiode and Shiode [32] detect point agglomerations at multi-scales by finding neighbor elements fallen within a certain shortest path distance from a reference point. Miller [33] shows that the Euclidean distance tends to overestimate the spatial intensity level of network-constrained features. Mennis and Guo [34] conclude that route distance is an effective measure to detect spatial clusters in network space. To the best of our knowledge, however, there is lack of network-constrained co-location scoping research that can be used for cases with more than two variables.

The classic co-location models employ the counting-based measures such as *support*, *confidence*, and *participation index*, which simply count the number of events falling in each cell to reflect the local interestingness [1, 23]. In contrast, spatial correlations with different distances have different weights in real-world data sets—the so called “Distance Decay Effects”. Since distance-decay effects exist in most of spatial processes as suggested by Tobler’s *first law of geography* [14], it is integrated into numerious geographical models for capturing the nature of spatial interactions. For example, the kernel function uses distance-decay effects to estimate the density variation of point clusters across space. Kernel density estimation is effective for the analysis of first-order properties (e.g., intensity), while spatial interactions between multiple variables need to be explored by other methods, e.g., cross *K*-function. In fact, in addition to the kernel density estimation, there are also many other successful models and applications which explore the distance-decay effects hidden in the spatial processes. For example, McGrail and Humphreys [35] measured the attractiveness of a facility service by materializing decay effect through using a gravity model. Liu et al. [36] found that the spatial interactions of inter-urban trip were governed by a distance decay effect following a power law distribution. Yamada and Thill [7, 31] incorporated the distance-decay for their network-constrained statistics involving single feature. More recently, Wang et al. [37] proposed a local co-location quotient based on statistical test. They differentiated the Euclidean distance and network distance for detecting co-location behaviors involving facilities and crimes. However, these approaches are developed from perspective of the spatial statistics, and as suggested in Huang et al. [20], they cannot be easily extended to deal with the co-location involving more than two features. To solve the problems above, our previous works have established a method which can extract prevalent co-location patterns from the whole study area (i.e., from a global scale), but they would also miss some valuable information existed within local areas [38,39]. In this respect, this paper proposes a new co-location scoping approach (Section 3) to identify prevalent regions of specific co-location patterns, which are pruned by previous approaches due to their low prevalence measure values at the global scale. Although the distance-decay effects are used in our study only for co-location patterns, it would be beneficial to incorporate this concept into other data mining frameworks (which are originally designed for handling transaction database) in order to improve their applicability for geographical settings.

## 3. Method

In the proposed method, each geographic feature can lead to a tessellated network (i.e., a linear tessellation) with density attribute, and multiple features will create different tessellated networks, which are further integrated to create a final network with co-location prevalence attribute. This scoping process involves three distinct stages:

**Step 1 Partitioning network:** Partitioning the street network into 1-D cells and, creating multiple tessellated networks for different features.
**Step 2 Quantifying “Distance Decay Effect”:** Calculating the distribution intensity of events on the tessellated networks by using a distance-decay function.
**Step 3 Interestingness evaluation:** Combining the tessellated networks into a composite network by using a weighted interest measure.

In the following, the method will be demonstrated in detail.

### 3.1 Partitioning network

As for vehicle navigation services, the concerned region often only needs to contain the segments and nodes which form the paths to the facilities of interest (e.g., hotel). Therefore, interesting regions for co-location patterns in our research are simplified as network paths, instead of the whole plane.

**Fig 2:**
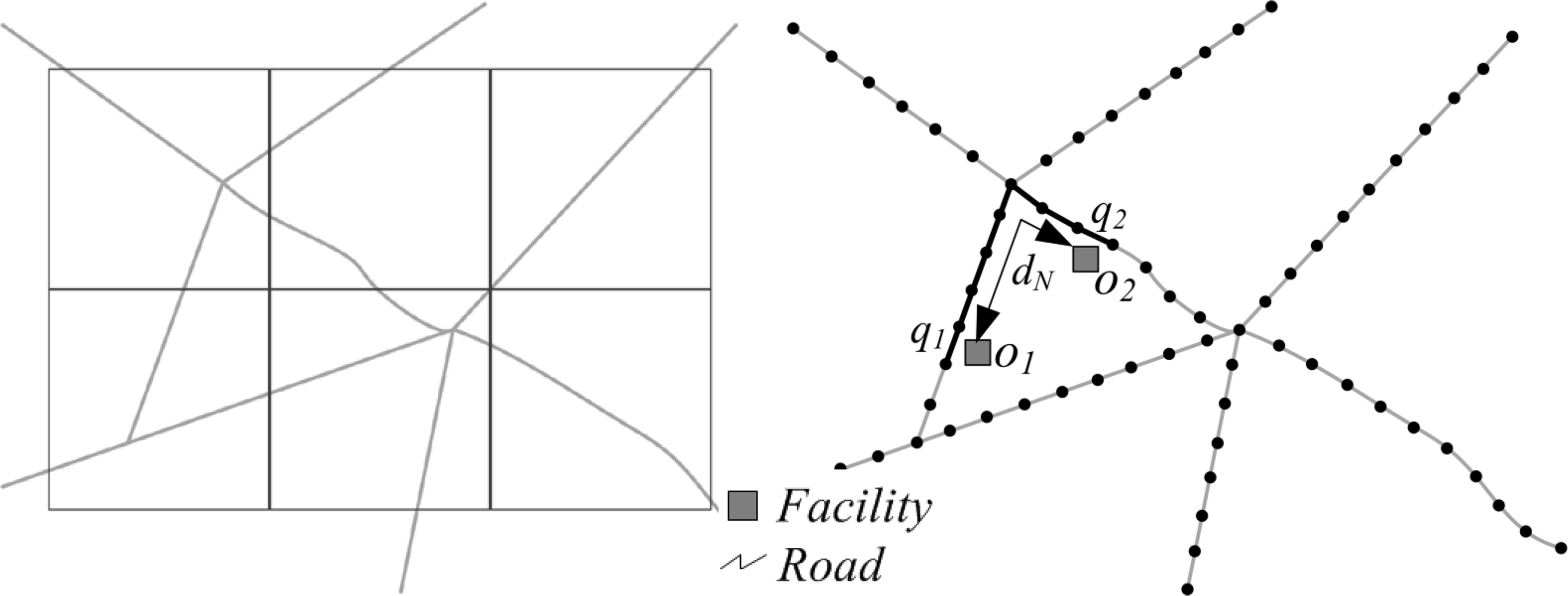
Two kinds of methods for partitioning a network into a grid: Using 2-D square cells (left), and 1-D linear cells (right). In the linear tessellation (right), two events *o*_*1*_ and *o*_*2*_ are projected to the nearest 1-D cells *q*_*1*_ and *q*_*2*_ by nearest distance searching, in order to facilitate the network analysis.

For determining co-location scope, the study region is firstly divided into a set of regular cells, which are often represented in the form of a 2-D square. While for the cases in a network-configured space, our method proposes to use a new form of cell with a unique, linear shape following the irregular configuration of networks, as shown in Fig 2. To obtain such type of cells, we firstly place a set of points (illustrated by the dark black points in Fig 2) which are set apart at a certain distance on the network. Then the street network is divided into small sections (i.e., 1-D cells) which start and end at a point. The size of these cells depends on the spacing distance between two neighboring points along the network. Within such network-constrained cell system, the unique spatial structure of network which may be lost in the 2-D cell system, can be maintained by establishing the topological relationship of the linear cells. Besides, in order to facilitate the network analysis, each event located alongside the network can be projected to the nearest 1-D cell (termed as *event cells*) according to its Euclidean distance from the central point of the cell. Then based on the locations of *event cell*, the neighborhood can be specified as the following:

#### Definition 1 (neighborhood)

Two events (*o*_*i*_, *o*_*j*_) is called neighborhood if the distance of the shortest path from the *event cell* of *o*_*i*_ to the *event cell* of *o*_*j*_ (i.e., d_N_(*o*_*i*_, *o*_*j*_)) is less than or equal to a user specified threshold.

In the linear tessellation, we define the network distance between two events as the number of hops of the cells on the shortest path that connects their projected *event cells*. Formally, network distance d_N_(*o*_*x*_, *o*_*y*_) between events *o*_*x*_ and *o*_*y*_ is calculated as:

$$d_{N}(o_{x},\;o_{y})=|\left\{q_{1},\;q_{2},\cdots ,\;q_{n}\right\}|-1$$ (1)

where {*q*_*1*_, *q*_*2*_, …, *q*_*n*_} denote the continuous cells on the shortest path connecting the projected cells of events *o*_*x*_ and *o*_*y*_, and |{*q*_*1*_, *q*_*2*_, …, *q*_*n*_}| denotes the number of the cells. As shown in Fig 2, events *o*_*1*_ and *o*_*2*_ are firstly projected to their nearest cells *q*_*1*_ and *q*_*2*_, respectively. Then with shortest-path searching, the cells located on the shortest path between *q*_*1*_ and *q*_*2*_ are identified (illustrated by the dark black lines in Fig 2), and finally according to Eq 1, the distance between events *o*_*1*_ and *o*_*2*_ is calculated as 7, which is the number of hops on the network shortest path, instead of their Euclidean distance.

### 3.2 Quantifying “Distance Decay Effect”

**Fig 3:**
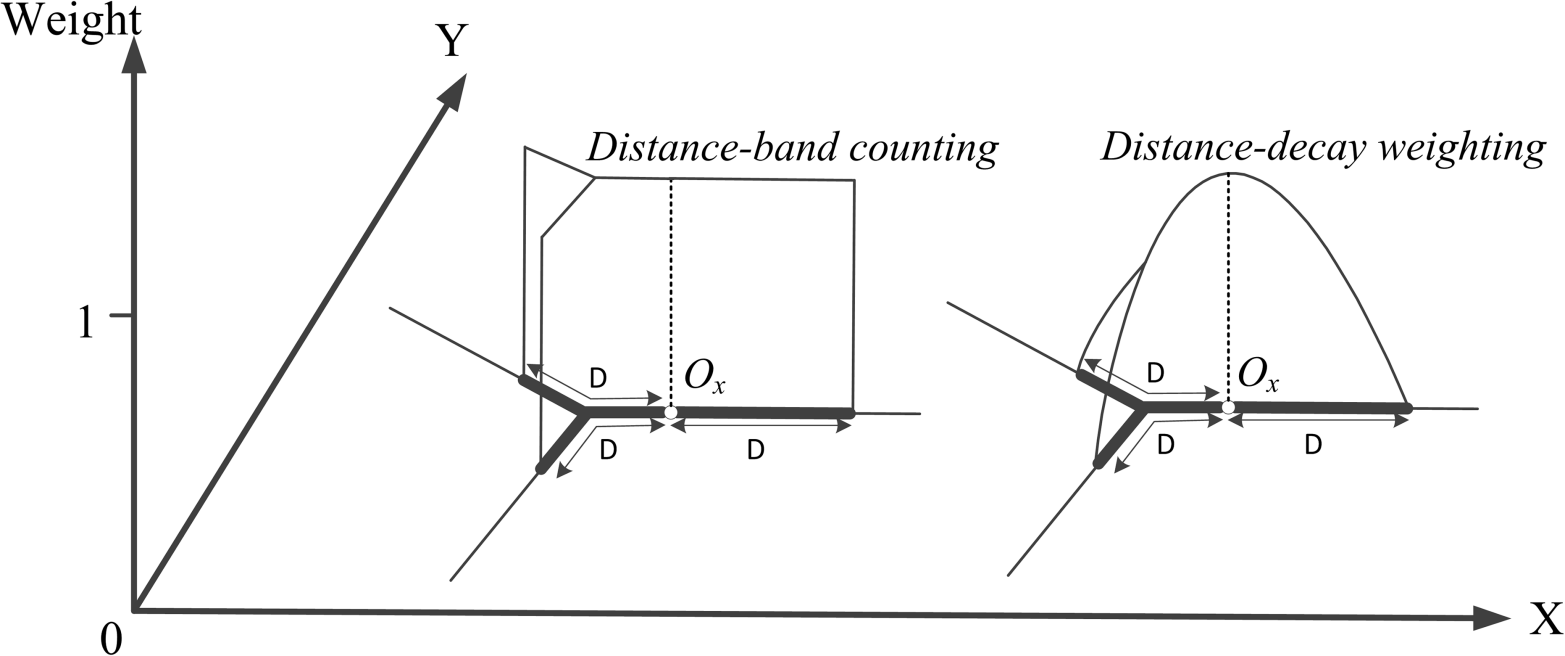
Comparison of the weighted functions based on distance-band counting and distance decay effect along a network.

“Distance Decay Effect” is applied in numerous applications involving the concept of spatial interaction, since the interaction between geographical events often take effect in a limited scope and its strength degrades with the increasing of distance [40–41]. Therefore, for identifying the scope of co-location patterns, we also propose to exploit the distance-decay function, which is inversely related to distance, to compute event intensity on the locations/cells of a network. As shown in Fig 3, rather than giving a constant weight to all locations within the neighborhood *D*, the distance-decay function introduces possible differentiation of proximity within the neighborhood boundary. This function would ensure that location close to the event (*o*_*x*_) has a large density value, whilst location far from the event has a small density value.

**Fig 4:**
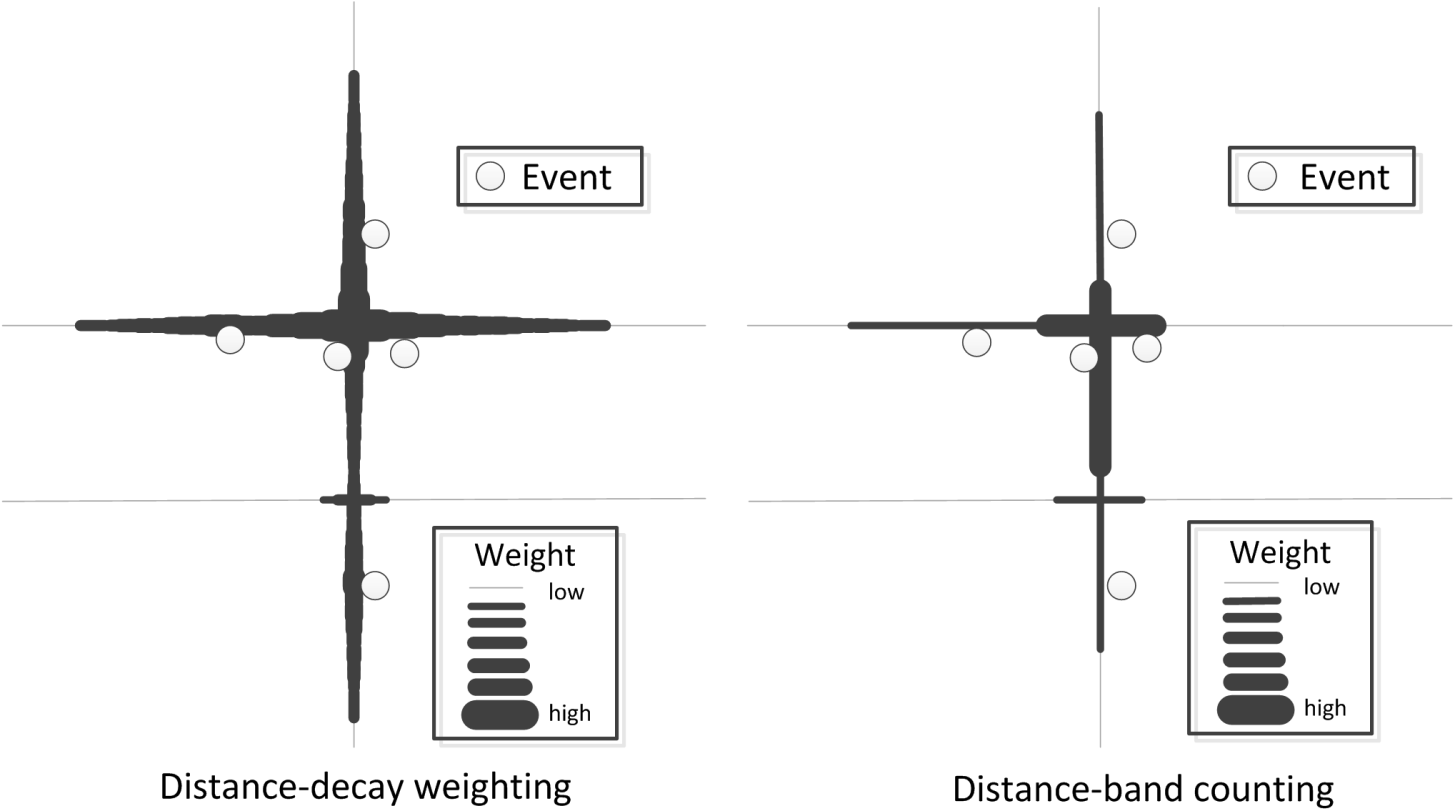
Illustration of the differences between the distance-decay measure and counting-based measure for the same point event dataset.

If a cell *q* falls within the neighborhood of multiple events (assuming the number of neighboring events is *n*), its density (Num(*q*, *f*_*i*_)) is the cumulative value of the distance-decay densities computed from all neighboring events which belongs to the feature type under consideration (*f*_*i*_), and the formation is as follows:

$$\mathrm{Num}(q,\;f_{i})=\sum_{x=1}^{n}S∙\frac{1}{D}∙(1-\frac{{\left(d_{N}(q,o_{x})\right)}^{2}}{D^{2}})$$ (2)

where *q* is the focus location/cell on the network, *f*_*i*_ the feature type under consideration, *o*_*x*_ the observed event (the feature type of *o*_*x*_ is *f*_*i*_), *S* the scaling parameter, *D* the theshold of neighborhood distance, d_N_(*q*,*o*_*x*_) the shortest-path distance between *q* and *o*_*x*_. An example with multiple data points is presented in Fig 4. Compared to the distance-band counting, the addition of a distance-decay function can avoid the problem of artificially sharp boundaries between neighboring cell values by generating a smooth density surface. Many different forms of the distance-decay function have been developed in the literature, including Linear, Gaussian, Conic, Quartic, and negative exponential [42]. It is approved that the choice of the distance-decay function does not affect much of the resultant density pattern, because they all account for “Distance Decay Effect” [42–44]. Therefore, for simplicity, this paper only considers the Quartic function (i.e., Num(*q*, *f*_*i*_)) with *S* equal to $\frac{3}{4}$, one of the most commonly used functions in geospatial domain [43]. Furthermore, in order to evaluate the probability of different features to frequently locate together, multiple networks with density attribute for different features will be generated in our approach by using the distance-decay function Num(*q*, *f*_*i*_).

### 3.3 Interestingness evaluation

In this section, we propose to integrate multiple density surfaces into a composite network by using a weighted measure called PI’(*q*) and formulate co-location pattern scoping in terms of this new concept. The composite network is termed as network of *Interestingness of Co-location*, or *IC* network for short.

**Fig 5:**
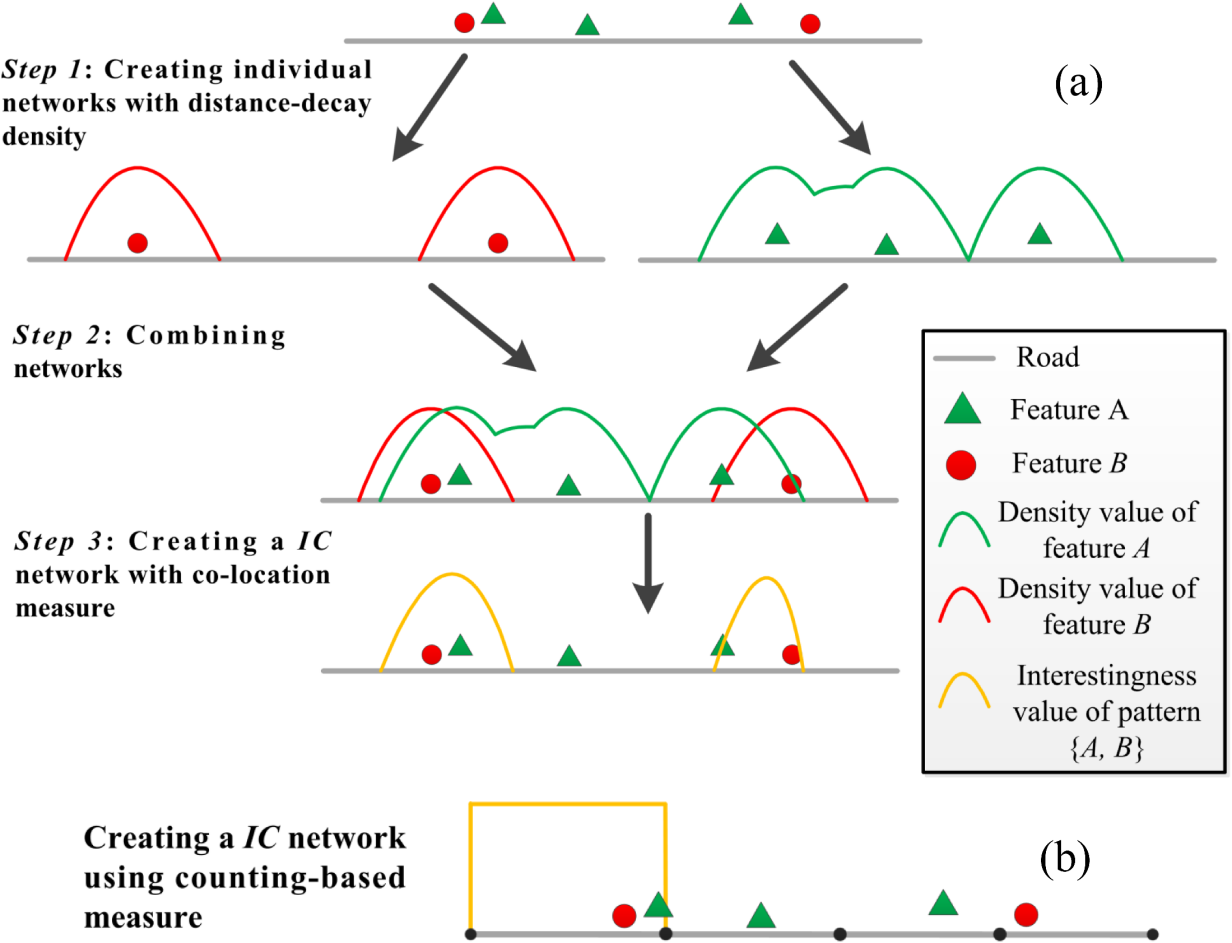
Process of calculating the interestingness variation of the co-location pattern {*A*, *B*} on an illustrative network: (a) using distance decay-based measure PI’(*q*), (b) using counting-based measure PI(*q*).

The authority measure evaluates the co-location interestingness by considering events falling within each individual cell [4]. Accordingly, we generalize the form of the measure through considering event distribution across adjacent cells. To demonstrate the advantages of using the weighted measure for co-location scoping, let’s consider a simple example. In Fig 5, five events classified into two categories (feature *A* and feature *B*) are distributed along an illustrated network segment. The detailed steps are as follows (Fig 5A):

1. In the first step of our approach (Step 1), a tessellated network with density attribute (i.e., Num) is generated for each feature, and a total of two tessellated networks for pattern {A, B} are in Fig 5A. The tessellated network with small cell size can lead to a smooth density surface, which is able to reduce the loss of spatial co-location information across the boundaries of cells.
2. Then, by utilizing the GIS *map overlay* technique (Step 2), these networks are combined into a final *IC* network (Step 3), on which the interestingness values of a co-location pattern *P* = {*f*_*1*_, *f*_*2*_,*…*, *f*_*k*_} are calculated using the improved *participation index* measure [10] as follows:
  $$\mathrm{PI}’(q)={\mathrm{Min}}_{f_{i}\in P}\{\mathrm{PR}’(q,\;f_{i})\}∙\mathrm{size}(q)$$ (3)
  where *q* is the focus cell on the network, PR′(*q*, *f*_*i*_) is the *participation ratio* value calculated by ${PR}^{\prime }(q,f_{i})=\frac{Num(q,f_{i})}{n_{f_{i}}}$ ($n_{f_{i}}$ is the number of events of feature *f*_*i*_ located in the neighborhood of cell *q*), size(*q*) is the regional data size of events in cell *q* calculated by $size(q)=\sum_{i=1}^{k}Num(q,\;f_{i})$. In the proposed model, the measure PI’(*q*) is not only impacted by the number of co-location instances, but also by the regional data size (size(*q*)) within each cell, because given the same *participation ratio* value, regions having more events are more statistically significant than regions having less events. A high value of the PI’(*q*) indicates that spatial features of the co-location pattern appear together in the cell *q* with high probability. In other words, a cell is a valid (or prevalent) place for the co-location pattern if all the features of the pattern are observed in the cell with a high probability of PI’(*q*). Compared to the counting-based measure *PI* (Fig 5B), our measure allows smooth estimate of the event density on the network (Fig 5A), and thus creates a more distinguishable result of co-location scoping. For example, by using the traditional measure, the right side of the network is obtained as a non-prevalent region for the pattern {A, B} because the events are located in different cells. However, this portion of the network is likely to be interesting according to the proposed measure PI’(*q*) (see Step 3 in Fig 5A).

Furthermore, it can be observed that replacing PR′(*q*, *f*_*i*_) with counting-based *participation ratio* in Eq 3 gives traditional measure PI [4, 10]. Therefore, the distance-decay interest measure can be considered as a generalization of counting-based interest measure, which takes into account the unique characteristics of spatial interaction that its strength bears a process of distance decay.

### 3.4 Algorithm analysis

For the task of co-location pattern scoping, our work proposes to improve the traditional algorithm [4] by using the network distance, instead of Euclidean distance. Thus, the performance difference between the proposed algorithm and traditional algorithm depends on the complexity difference in computing spatial proximity. Specifically, two major operations in our framework influence the computational cost, i.e., partitioning the space by using 1-D cells and computing the distribution density of events by applying the shortest-path searching technique. Given the number of street edges *n* and the number of 1-D cells *l*, the computational complexity of the linear tessellation operation is O(*nl*). For the calculating of spatial density of events, the time cost depends on the efficiency of calculating network distance. In general, the shortest-path searching can be implemented by the Dijkstra [45]’s algorithm, which adopts a strategy of incrementally expanding the network around the query event. Thus, for a specific feature, if its number of events is *m*, the run time complexity of calculating the density of events is O(*mlk*^2^) [45], where *l* is the number of 1-D cells and *k* is the number of nodes on the network. Therefore, given the number of features *p*, the overall run time complexity of calculating the interest measure values is O(*pmlk*^2^). Based on the above, our algorithm has the time complexity O(*nl*)+O(*pmlk*^2^), which is higher than the traditional algorithm that only requires a constant-time operation for calculating Euclidean distance. However, our algorithm is more accurate for some specific cases involving network-constrained features (e.g. urban facility).

In addition, there are three key issues that should be paid attention to in our algorithm. Firstly, as presented in Section 3.1, the method tessellates a street network by placing equidistant points on the network, and thus the running time of this step would largely depend on the granularity of cell size. Generally, the smaller the interval between points, the more cells the algorithm traverses, and thus the more time it costs. Therefore, although the step of partitioning network has a time complexity of O(*nl*), it usually takes much time in practical applications because the number of cells is usually large in the implementation. In this respect, the cell size should be carefully defined according to data distribution. For example, our case study used a parameter of 10 m (please see Section 4.2), which can ensure both effectiveness and efficiency of the proposed method. Secondly, although corner side cells with a length less than the predefined length may be generated in practical conditions, they would not affect the result due to the following reasons: (1) Corner side cells with a short length only account for a negligible portion of all the cells; (2) As long as the linear segment size is small enough, the resolution difference between regular cell and corner side cell can be significantly reduced. Thirdly, since travel speed varies among different streets in reality, the neighbourhood size should be defined differently depending on the local traffic condition. The proposed method can be adapted to this situation by introducing irregular sizing cells. The basic idea is to assign a big size of cell to the street segments having a high travel speed and a small size of cell to the street segments having a low travel speed. In this way, with additional traffic data sets (e.g., vehicle trajectory), it is possible to deal with the more complex of data using the proposed framework.

## 4. Experiments and results

### 4.1 Experiment data

**Fig 6:**
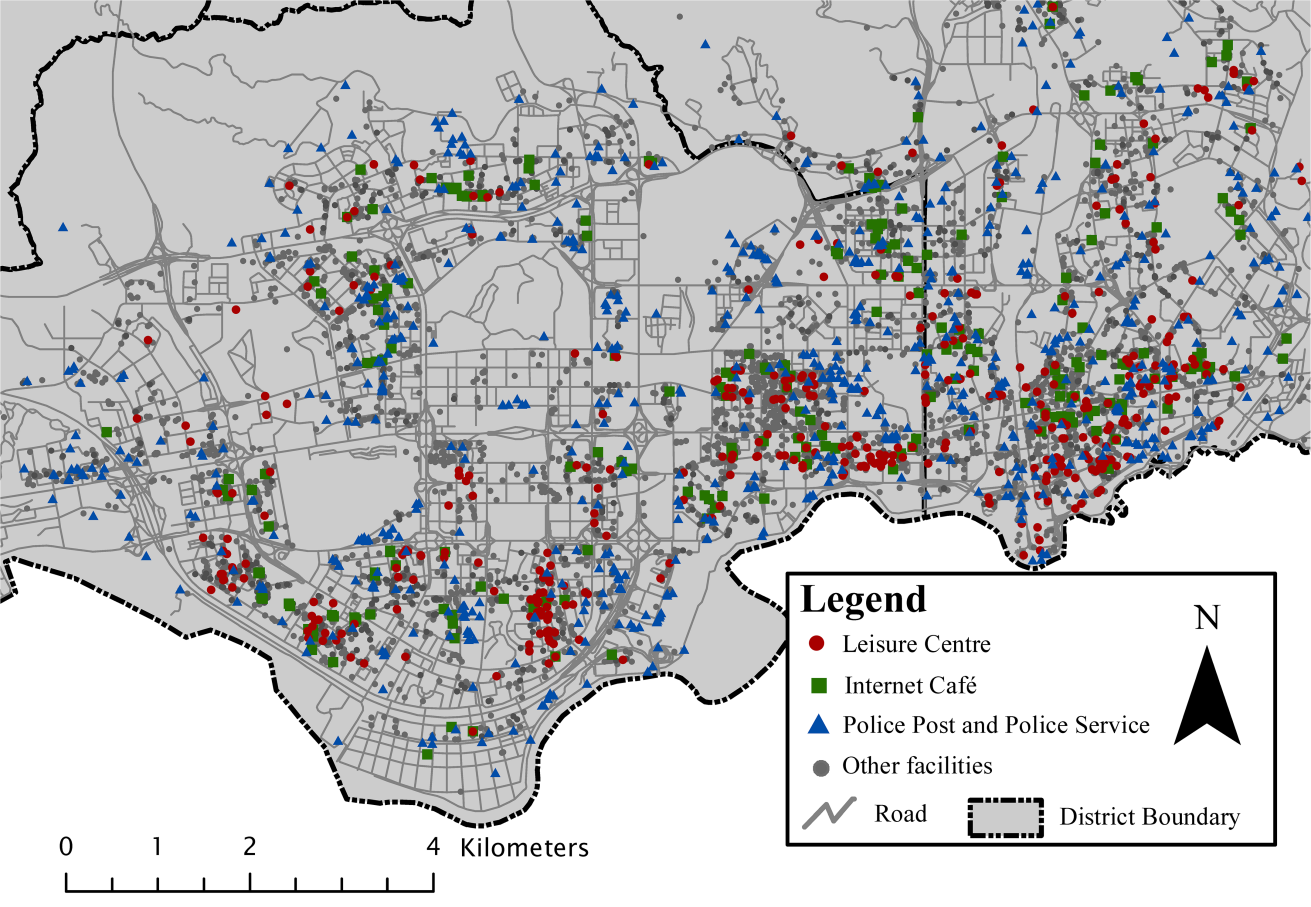
Study region, the facilities and the street network data.

The data sets were provided by the Geomatics Center of Shenzhen (http://www.szpl.gov.cn/), including a street network and a set of urban facility POIs (Points of Interest) from Shenzhen, China, as shown in Fig 6. In our previous work (please see [38]), we chose sixteen certain types of facilities for the global co-location pattern mining. Among the non-prevalent patterns in the global region, we selected two patterns for the regional co-location scoping, i.e., for identifying local areas in which the selected patterns are significant or prevalent enough. The two regional patterns include the size-2 pattern {*Internet Café*, *Police Post and Police Service*} and the size-3 pattern {*Internet Café*, *Leisure Center*, *Police Post and Police Service*}. The proposed method can also be applied to other patterns with different features and pattern sizes. The underlying street network possesses 4176 segments, which are split at the locations of street intersections.

### 4.2 Experiment settings

In our experiments, the neighborhood threshold was set to 300 m for two reasons: firstly, by constructing street blocks from the street network in Shenzhen, we found that the average block neighborhood is about 228 m; In addition, researchers [46] in urban studies suggested that 300 m distance is suitable for the analyzing of spatial interactions at the scales of neighborhood. Furthermore, in order to evaluate the effectiveness of our approach at different distance scales, larger distance thresholds were additionally utilized in the co-location scoping, i.e., 600 m and 1200 m.

For the identifying of valid locations of the co-location patterns, six experiments were conducted (Table 1). The first two experiments were based on measure PI’(*q*). For both of the two experiments, neighborhood threshold (300 m) were the same, and the basic linear cell size was set to 10 m. In essence, in our approach, the size of cell should keep as small as possible to generate smooth density surface and facilitate co-location analysis on different locations. However, cells with small size lead to a rapid increase of amount of data, which makes the algorithm efficiency being a problem in the experiments, especially when the experiments do not require such a small granularity. After several tests on the experiment data, we chose to partition the network with an appropriate parameter (10 m), which is small enough relative to the average length of the street segments in the data. To compare our approach (Experiments I and II) with the traditional approach [4], two additional sets of experiments (Experiments III and IV; Experiments V and VI) were also conducted. These experiments differ from Experiments I and II in that, the sizes of the basic cells (the length of network segments and 300 m) were larger than that in Experiments I and II (10 m). This is because counting-based interest measure considers events in each cell as a co-location instance, ignoring relationships across the boundaries of cells. Therefore, a large cell size parameter should be used in traditional approach for reducing the loss of co-location information. In addition, Experiments V and VI adopted the classic 2-D grid and measured spatial proximity using Euclidean distance, while Experiments III and IV adopted a 1-D grid by dividing the street network on the locations of street intersections.

**Table 1:** The six experiments for the study region and their settings.

Experiments	Regional co-location pattern	Tessellated space	Neighborhood determination	Interestingness measure
I	{*Internet Café*, *Police Post and Police Service*}	1-D grid (10-m units)	network distance (300-m threshold)	Distance decay-based participation index PI’(*q*) (Quartic function)
II	{*Internet Café*, *Leisure Center*, *Police Post and Police Service*}	1-D grid (10-m units)	network distance (300-m threshold)	Distance decay-based participation index PI’(*q*) (Quartic function)
III	{*Internet Café*, *Police Post and Police Service*}	network segments	network distance (300-m threshold)	Counting-based participation index PI(*q*)
IV	{*Internet Café*, *Leisure Center*, *Police Post and Police Service*}	network segments	network distance (300-m threshold)	Counting-based participation index PI(*q*)
V	{*Internet Café*, *Police Post and Police Service*}	2-D grid (300-m squares)	Euclidean distance (300-m threshold)	Counting-based participation index PI(*q*)
VI	{*Internet Café*, *Leisure Center*, *Police Post and Police Service*}	2-D grid (300-m squares)	Euclidean distance (300-m threshold)	Counting-based participation index PI(*q*)

### 4.3 Results and discussion

In general, according to our previous work [38], the most prevalent co-locations involve frequent spatial features (e.g., *ATM*, *Parking Lot*) that have far more events than others. This is because from the perspective of the entire study region, more frequent features are more likely to appear in different locations, and thus they have a higher probability to co-occur with others than rare features. For example, the two patterns of interest involving rare feature *Internet Café* obtain a lower participation index value (Table 2), and they could be pruned by a prevalence threshold in the global co-location mining. To solve this limitation, we applied the proposed approach to identify local areas in which the patterns of interest were significant enough (Section 4.3.1).

**Table 2:** The patterns of interest and their prevalence measure values in the entire study region with neighborhood distance D = 300 m.

Pattern size	*Co-location*	Global interest measure value (participation index)
Size-2 pattern	{*Internet Café*, *Police Post and Police Service*}	0.191842
Size-3 pattern	{*Internet Café*, *Leisure Centre*, *Police Post and Police Service*}	0.068440

#### 4.3.1 Regional co-location pattern scoping

**Fig 7:**
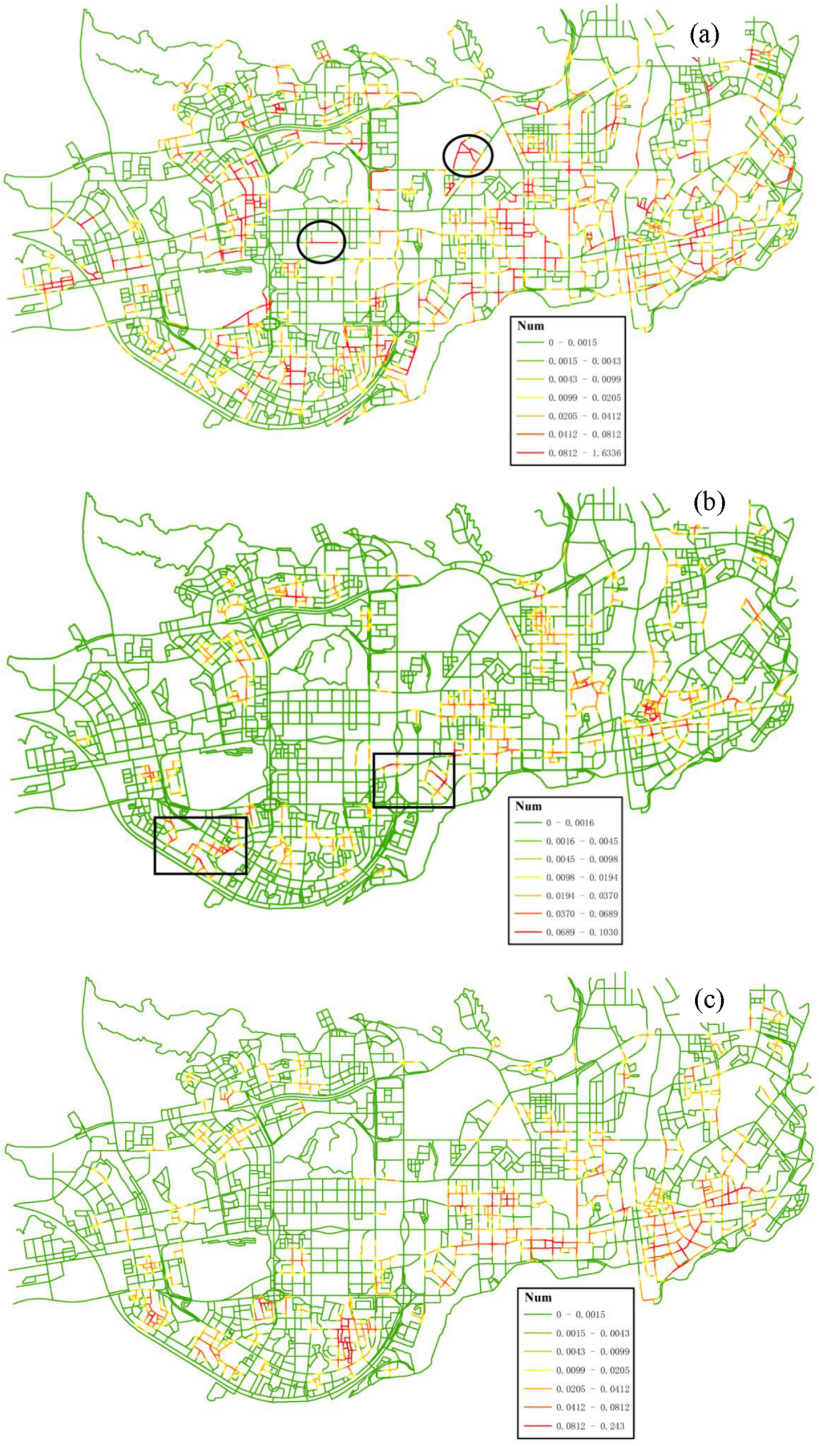
Tessellated networks with density attribute (Num) generated from the data of different types of features: (a) *Police Post and Police Service*, (b) *Internet Café*, (c) *Leisure Center*. All the density values are calculated with a Quartic function and 300-m neighborhood.

By implementing Experiments I and II, three tessellated networks with density attribute were generated for three features respectively (Fig 7). Peaks of the density networks (highlighted by red color) represent the presence of *hot spots* in the event distribution. By a simple visual observation, it can be noticed that the association between the three types of facilities shows huge variation across the study area. The main clusters of these features appear in the southern part of the region, adjacent to Hong Kong in the real world. Geographical advantages of the local area facilitate the development of social-economic activities. In addition, it is also observed that some *hot spots* in Fig 7A (highlighted by the circles) do not appear in Fig 7B, while a contrary trend is observed in other regions highlighted by the squares in Fig 7B.

**Fig 8:**
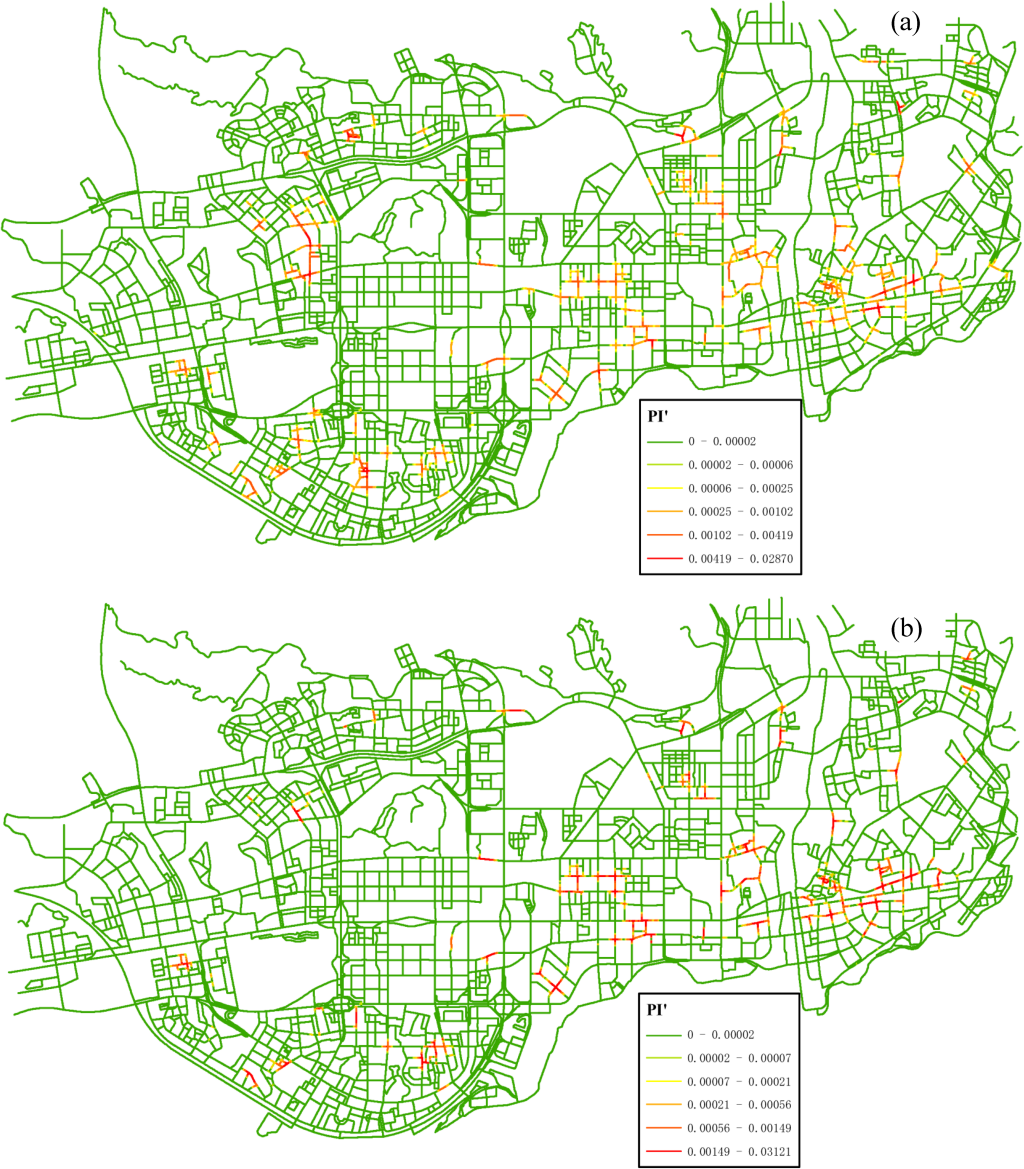
*IC* networks with distance-decay interestingness attribute (PI’) generated for different co-location patterns: (a) {*Internet Café*, *Police Post and Police Service*} (Experiment I), (b) {*Internet Café*, *Leisure Center*, *Police Post and Police Service*} (Experiment II).

In order to quantify these local variations in co-location scoping, we further integrated the density networks into a composite *IC* network using the proposed measure PI’(*q*). Fig 8 shows the detailed distribution trends of co-location interestingness values. First, for the size-2 pattern {*Internet Café*, *Police Post and Police Service*}, most of high measure values with a clustered pattern are located in the southern and eastern parts of the study region. In contrast, most of low measure values are distributed in specific areas satisfying either one of the following conditions: (1) both of *Internet Café* and *Police Post and Police Service* features have a low density of events; (2) one of the features has a low density of events (e.g., the locations highlighted by the circles in Fig 7A and by the squares in Fig 7B). Actually, this also occurs with the size-3 pattern; That is, the high interest measure values are clustered in the south-east parts of the study region (Fig 8B). In general, the interestingness surface for the size-3 pattern is massively diluted and reduced in comparison with that for the size-2 pattern (i.e., Fig 8A vs. Fig 8B), because the addition of the third feature (*Leisure Center*) makes it harder for the co-location pattern to be significant in a local area.

With such clear clustering information associated with police presence and other facilities, understanding where and why to increase police presence is possible to creating a safe urban environment. Street crimes which often occur on street are rarely random in space. Especially according to the reports from the government’s safety agency, the majority of street crimes and youth crimes take place in the nearby areas of the specific facilities (i.e., *Internet Café* and *Leisure Center*), because there are actually no effective measures to supervise the operation in these neighborhoods. Hence, our approach is deemed as a key issue in safety improvements, with identifying high-crime street segments.

#### 4.3.2 Comparison

**Fig 9:**
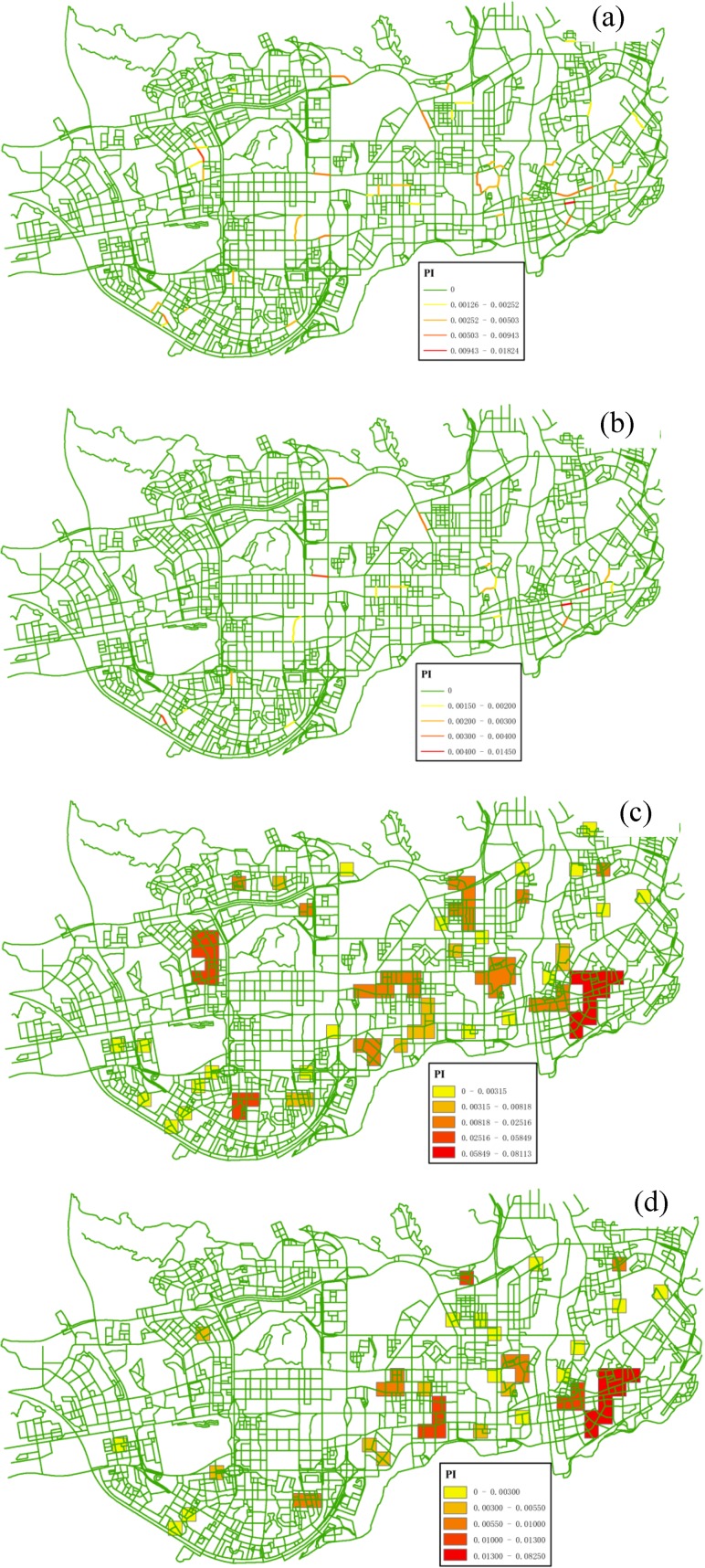
Distributions of co-location interestingness values based on the counting-based measure *PI*: (a) Experiment III, (b) Experiment IV, (c) Experiments V, (d) Experiments VI.

To compare our approach with the traditional approaches, the other four experiments (III-VI) were also implemented in the study region (Fig 9).

All of the experiments produce a very different result in co-location region discovery, including the scope of valid regions and the distribution of interest measure values. We can make the following observations from a more detailed comparison. First, for the comparison between Fig 8A and Fig 9A, what they share in common is they both limit co-location region to paths with 1-D cells. And the difference is that the valid paths in Fig 8A consist of continuous small linear cells, while the valid paths in Fig 9A consists of coarse-grained paths. Furthermore, the *IC* surface with distance-decay function shows a smoother characteristic compared to that with counting-based function. In Fig 9A, many examples of artificially sharp boundaries at the locations of street intersections exist. This trend can also be seen when we compare Fig 8B and Fig 9B for the size-3 pattern. The problem of the use of counting-based measure on co-location scoping is due to the following two reasons: the counting-based measure only accounts for co-location instances falling within a single cell, neglecting instances within its neighboring cells; and also the counting-based measure does not differentiate the proximity of locations within neighborhood. On contrary, our approach considers distance-decay propagating characteristic of spatial interaction through the space and hence provides a better solution for our cases.

**Fig 10:**
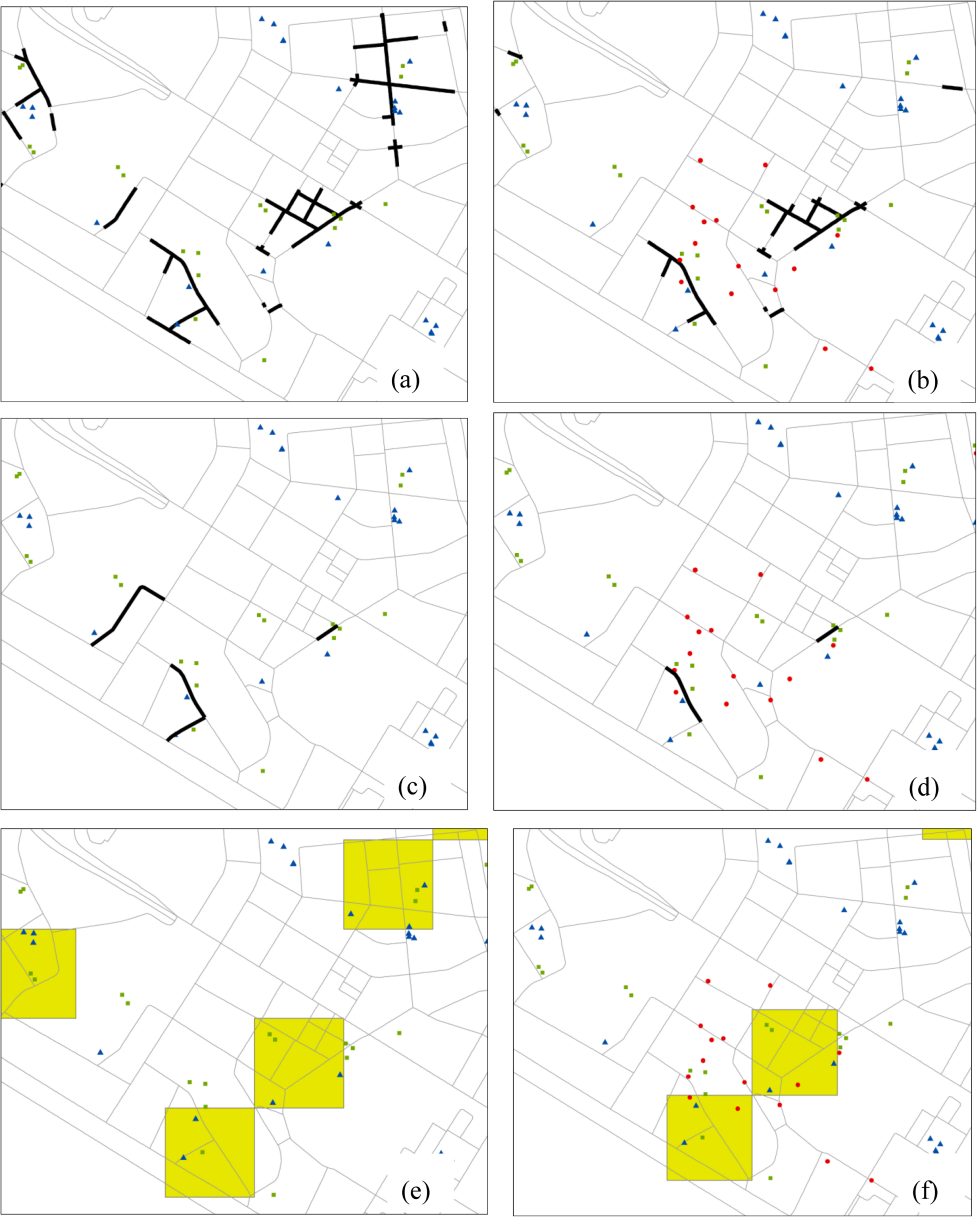
The illustration of valid paths (dark segments) or regions (yellow polygons) in a small portion of the Shenzhen city: (a) Experiment I, (b) Experiment II, (c) Experiments III, (d) Experiment IV, (e) Experiment V, (f) Experiments VI.

Second, Experiments V and VI result in several valid regions not only containing the network paths, but also the areas apart from the network routes. With the planar approach, facilities on a non-contiguous network section may fall within the same neighborhood, resulting in many discrete road sections for the valid regions of the patterns, as presented in a zoom-in portion of the valid segments (Fig 10). Experiments I and II which utilize network distance instead of Euclidean distance only consider contiguous network section for the modeling of spatial co-locations, and thus they give more reasonable results.

Third, Table 3 presents the resulting statistics. Among Experiments I-IV, all of which adopt 1-D grid, Experiments I and II with a distance-decay measure result in far more contiguous road paths (i.e., clusters for the co-location pattern) than Experiments III and IV do with a counting-based measure. This finding can be also observed in the comparison between Experiments I-II with network distance and Experiments V-VI with Euclidean distance. In summary, the statistics confirm the impression of the above mining result (Figs 8 and 9): that is, our approach can generate a large number of road paths that are spatially contiguous, avoiding sudden divergence at the boundaries of cells. These regions can then be utilized by domain experts for confirmatory analysis to help answer the *what* and *why* questions of the applications of urban management and transport planning.

**Table 3:** The number of valid cells and valid clusters whose PI’(*q*) >0 or PI(*q*) >0 for the six experiments.

Statistics	Experiments					
	I (*PI*_*w*_ >0)	II (*PI*_*w*_ >0)	III (*PI* >0)	IV (*PI* >0)	V (*PI* >0)	VI (*PI* >0)
The number of valid cells	9149	5438	42	20	81	46
The number of valid clusters	148	117	36	18	38	28

#### 4.3.3 Statistical test

Next, we computed statistical measure values of the results (Fig 8) to determine to what extent a co-location instance in a given cell conform to a particular hypothesis. Such statistical significances may be tested in different ways. A natural one is to examine to what extent a given set of features co-occur in a cell at a probability different to what one would expect. Since the *participation index* can measure the probability that the events of different features have co-location relationships [9], we can use the following measure to test the statistical significance:

$$\varepsilon \;(q)={\mathrm{Min}}_{f_{i}\in P}\frac{\mathrm{PI}’\;(q)-E(f_{i})}{\sigma (f_{i})}$$ (4)

where *q* is the cell under investigation, *P* = {*f*_*1*_, *f*_*2*_,*…*, *f*_*k*_} is the set of features of interest, PI’(*q*) is the *participation index* value at cell *q* containing events of all the features {*f*_*1*_, *f*_*2*_,*…*, *f*_*k*_}, E(*f*_*i*_) is the average *participation index* value at cells that contain events of feature *f*_*i*_, and σ(*f*_*i*_) is the standard deviation of the *participation index* values at cells that contain events of feature *f*_*i*_. In this case, E(*f*_*i*_) serves as a null hypothesis with respect to which we can measure PI’(*q*). The measure *ε*(*q*) is standardized to give the statistical significance in terms of σ(*f*_*i*_). According to the normal distribution, *ε*(*q*)>2 corresponds to the confidence interval of 95%. If the *ε*(*q*) is larger than 2, the assumption of independent distributions in the cell under investigation is rejected and a significant location has been found. There are other measures that may be used in this statistical test, such as statistical measure of the results versus complete spatial randomness. But, their running time increases fast with the increase of the number of instances and sizes of patterns, because they usually require a large number of times of the Monte Carlo simulations (at least 1000). Therefore, previous approaches in spatial co-location mining tend to use the measure of statistical diagnostics [47].

**Fig 11:**
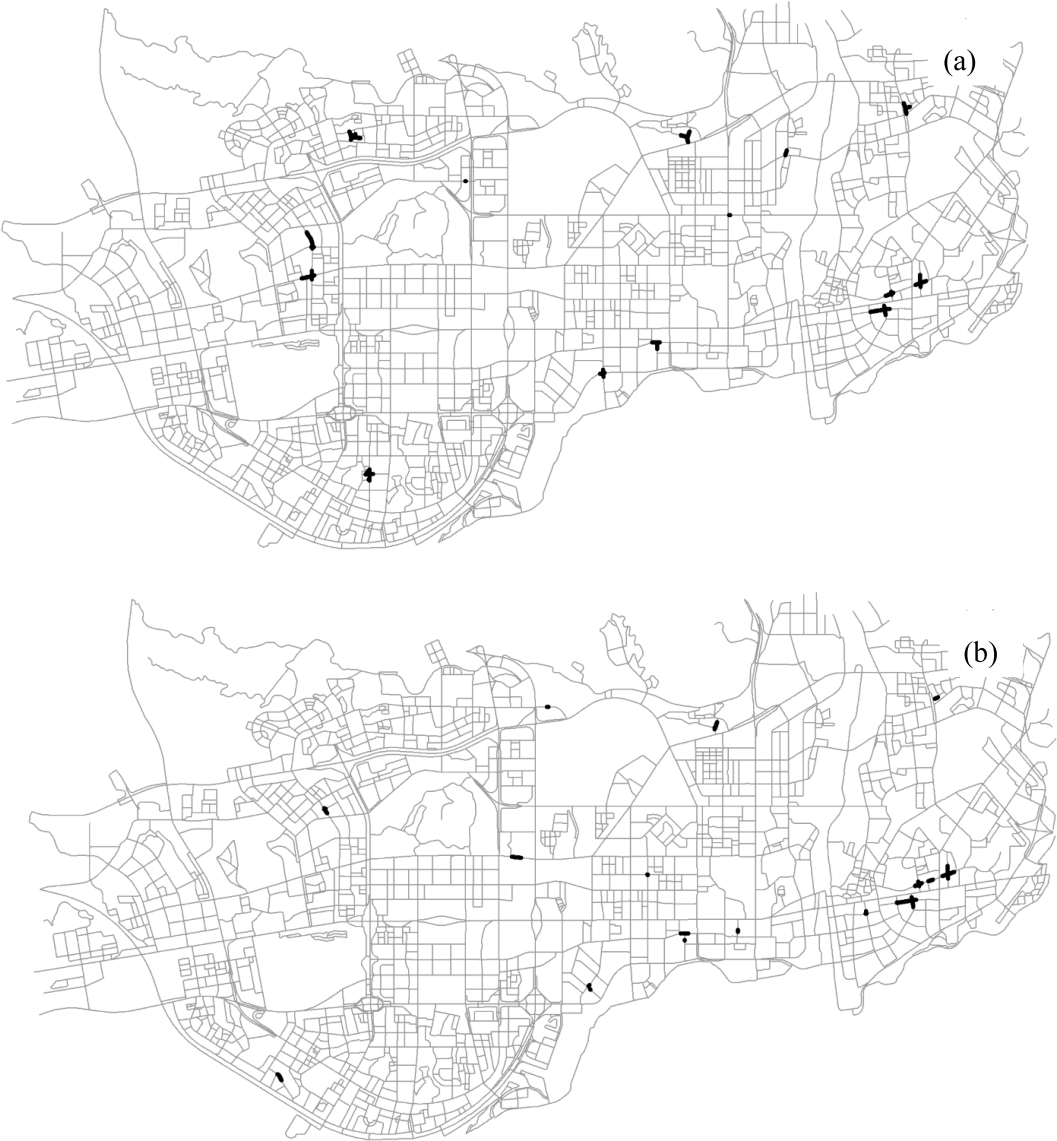
The significant segments (dark segments) for the two co-location patterns at the 0.05 significance level: (a) {*Internet Café*, *Police Post and Police Service*} (Experiment I), (b) {*Internet Café*, *Leisure Center*, *Police Post and Police Service*} (Experiment II). Both the statistical tests are conducted with a 300-m neighborhood.

By adopting the proposed statistical measure, we obtained the significant segments with respect to the patterns of interest, as presented in Fig 11. In these cells where *ε*(*q*)>2, the features of interest co-occur with a probability significantly larger than one would expect relative to the null hypothesis. The significant cells of both the size-2 pattern and the size-3 pattern are located in nearly the same areas, i.e., peripheral areas of the study region.

#### 4.3.4 Effect of neighborhood distance scale

**Fig 12:**
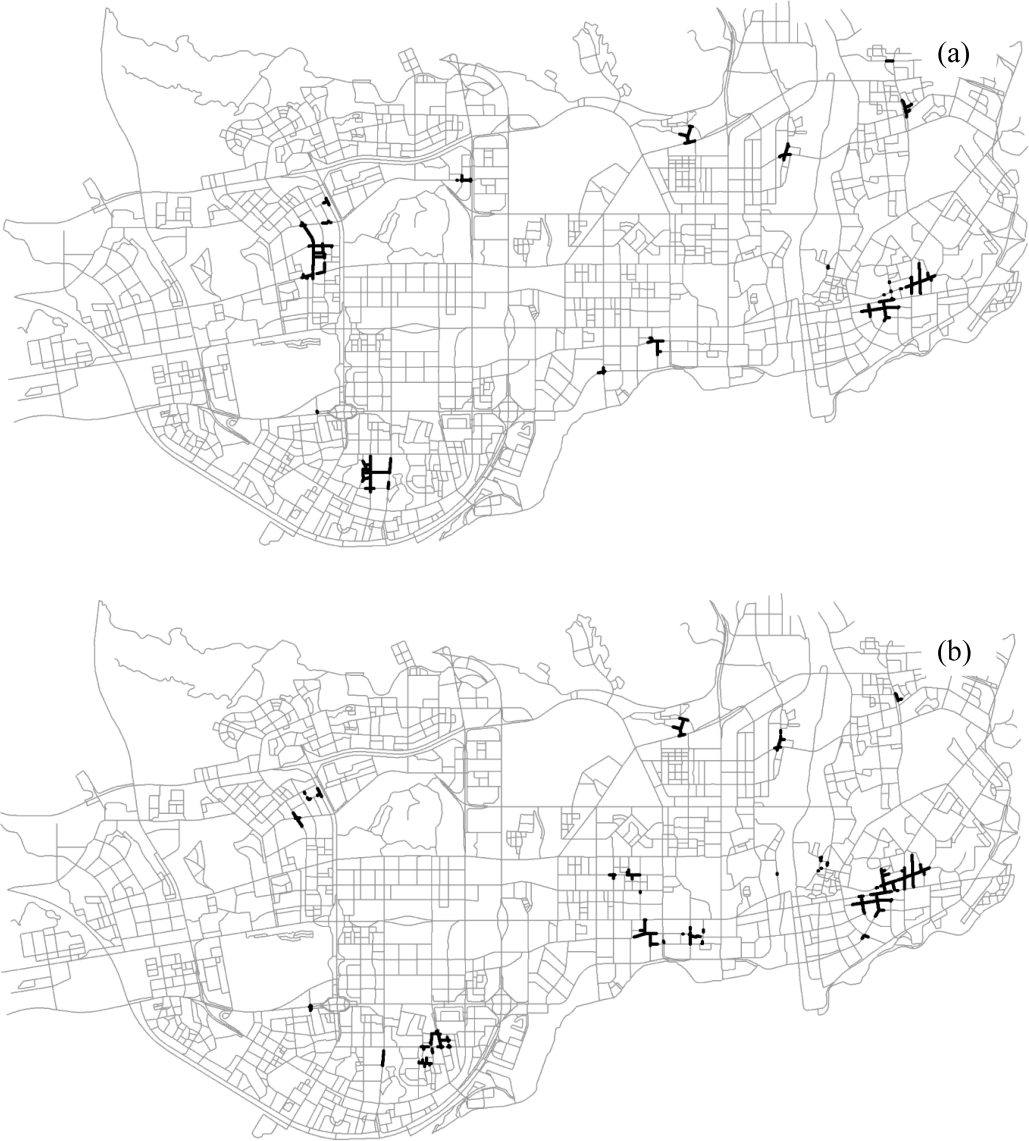
The significant segments (dark segments) for the two co-location patterns with a 600-m neighborhood: (a) {*Internet Café*, *Police Post and Police Service*}, (b) {*Internet Café*, *Leisure Center*, *Police Post and Police Service*}.

**Fig 13:**
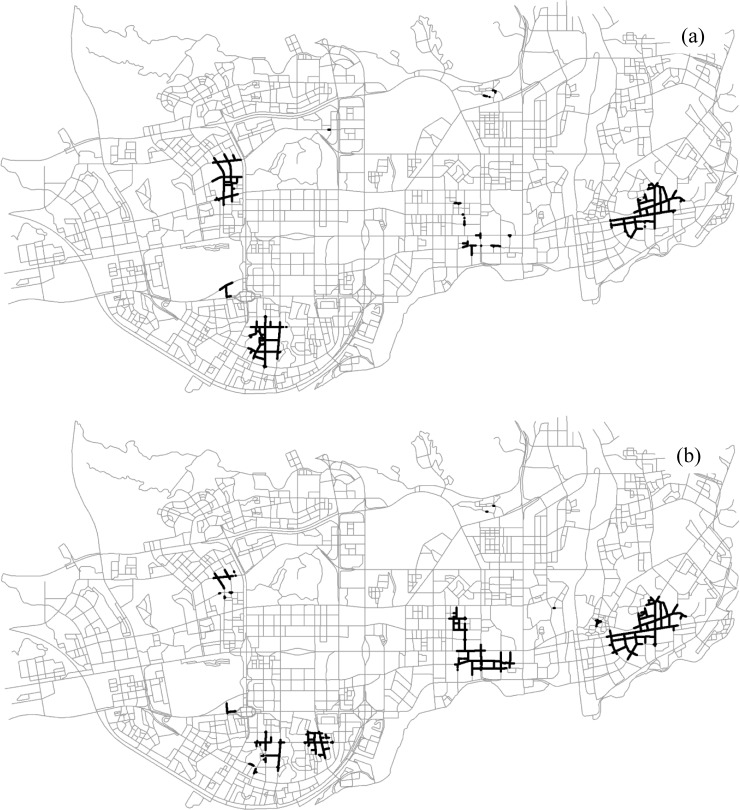
The significant segments (dark segments) for the two co-location patterns with a 1200-m neighborhood: (a) {*Internet Café*, *Police Post and Police Service*}, (b) {*Internet Café*, *Leisure Center*, *Police Post and Police Service*}.

We also examined the impact of neighborhood distance scale on the significance assessment and co-location region detection (Figs 12 and 13). As the neighborhood distance threshold increases (i.e., 300 m, 600 m, and 1200 m), the experiments result in larger significant regions for both the size-2 pattern and the size-3 pattern. It makes sense, since the significant regions detected with larger neighborhoods often indicate the existence of more extensive spatial interactions along the network. Furthermore, the results imply that the choice of neighborhood distance depends on the scale of analysis; that is, a small neighborhood distance will identify local significant regions for the pattern of interest, while a larger distance results in broader areas.

#### 4.3.5 Evaluation

**Fig 14:**
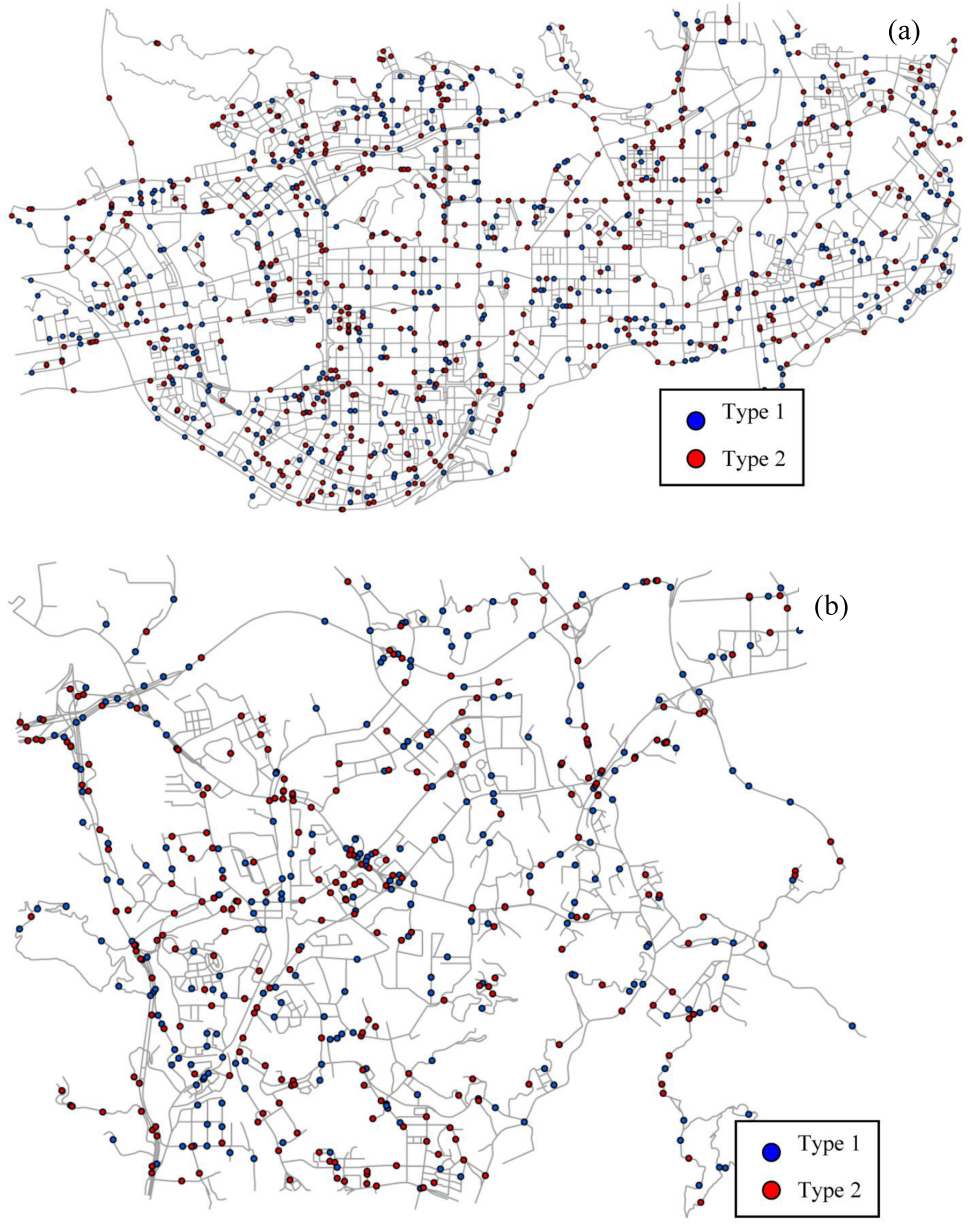
Random points along the network within (a) the highly urbanized area and (b) the suburban area.

In order to compare the effects of network distance and Euclidean distance on the co-location mining, we further generated two sets of random points along the networks within the highly urbanized area (Fig 14A) and the suburban area (Fig 14B). For each area, there are two types of simulated points which are independent and distributed uniformly over the network, and thus the size-2 pattern consisting of these two types is expected to be non-prevalent. In this respect, the lower the prevalence measure value obtained, the more accurate the method is. Table 4 presents the result for the urbanized area with high density network and the suburban area with low density network. It can be observed that the prevalence measure values based on network distance are always lower than those based on Euclidean distance. Therefore, the proposed method is more accurate in network space than traditional methods. Furthermore, as shown in Table 4, the difference between the prevalence measure values of network distance and Euclidean distance in the suburban area is larger than that in the urbanized area. It means that the effectiveness of network distance on co-location mining is more significant in the suburban area than in the urbanized area. This is because the network distance approximates a straight line distance (Euclidean distance) when the network density is increased.

**Table 4:** Comparison of the effectiveness of planar co-location mining and network co-location mining within the highly urbanized area (Fig 14A) and the suburban area (Fig 14B), by generating random points along the network.

Region	Planar co-location mining		Network co-location mining	
	Number of co-location instances	Prevalence measure value	Number of co-location instances	Prevalence measure value
The highly urbanized area	954	0.814000	507	0.614000
The suburban area	457	0.746212	246	0.518939

Finally, we compared the efficiencies of the proposed algorithm and the traditional algorithm with different neighborhood distance thresholds (i.e., 300 m, 600 m and 1200 m). Both of the algorithms can be divided into two procedures: neighbor pairs searching and pattern mining. As discussed in Section 3.4, the proposed algorithm spends more time in searching neighbor pairs than the traditional algorithm does. In addition, compared to network distance, Euclidean distance would result in more co-location instances, and thus the traditional algorithm would spend more time in pattern mining process. Table 5 presents the result of performance of both algorithms. It can be observed that, with 300-m neighborhood distance threshold, the overall computational time of the proposed algorithm is more than that of the traditional algorithm. But our algorithm performs better with the increase of threshold.

**Table 5:** Comparison of the computational time of planar co-location mining and network co-location mining with different neighborhood distance thresholds (i.e., 300 m, 600 m and 1200 m).

Neighborhood distance threshold (m)	Planar co-location mining			Network co-location mining		
	Neighbor pairs searching (s)	Pattern mining (s)	Overall computational time (s)	Neighbor pairs searching (s)	Pattern mining (s)	Overall computational time (s)
300	0.157	0.857	1.014	1.454	0.799	2.253
600	0.173	23.862	24.035	9.022	3.453	12.475
1200	0.212	274.339	274.551	113.191	10.605	123.796

## 5. Conclusions and outlook

Spatial co-location pattern mining approach has been widely used for discovering spatial knowledge from spatial data sets, and regional co-location scoping has been proven to be better for identifying interesting regions in which co-location patterns concerned are significant. These two approaches, together, can be used to model and analyze spatial data sets at different levels of granularity, which are global-grained one and regional-grained one. Yet, most of the previous research [4, 10] ideally abstract the study region as a homogeneous Euclidean space, computing spatial proximity by using Euclidean distances. In urban environment, however, the movements are usually restricted to the layout of street network and the patterns concerning spatial interactions largely depend on the computation of network distance instead of Euclidean distance. Given the importance of co-location scoping in network space, this paper proposes a novel method for identifying the valid regions of patterns involving urban facility POIs (Points of Interest), which can be also used in the spatial data mining for other network phenomena, such as traffic accidents and street crimes.

Unlike the previous approaches using square cells [4], our approach proposes to partition the study region into 1-D cells, which possess specific network characteristics. The tessellated data structure facilitates the calculating of network distance and the interestingness assessment of locations on a network. On the other hand, this paper quantifies the “Distance Decay Effect” of spatial interaction, which exists extensively within many spatial objects and spatial relations in the real world. With introducing the distance-decay function, a new measure PI’(*q*) is defined in the approach to give the interestingness of locations/cells with respect to the pattern under investigation. It differs from the traditional counting-based measure by producing smooth density surface and identifying valid regions with a spatially contiguous characteristic. Experimental results demonstrate that in comparison with traditional techniques our method is effective in dealing with network-constrained pattern scoping problems, which involve the propagation and decay of the strength of spatial interactions.

However, there are several aspects in which the proposed framework can be further extended. First, in addition to the street network, there are many other types of spatial networks in the real world, such as power lines and pipelines, the flows on which largely depend on the direction and connectivity conditions. Our approach however only considers the physical distance constraint, and further development can construct a directional graph with turn prohibition for the refining of neighborhood. Furthermore, it should be noted that the physical distance concept does not apply to the cases in completely non-spatial networks (e.g. social network or dependency chains). To solve this limitation, we can calibrate the model by assigning different weights to different network links according to the current context of the user in social network (e.g. current social relations and personal preferences). In this way, a ‘virtual’ distance concept instead of ‘physical’ distance concept can be developed in the framework for non-spatial networks.

Second, the proposed approach does not consider the constraint of facility difference. The service area and competitive capability largely depend on the attributes of facility, such as mall size and power. Thus, the further development of the model should add a weight coefficient in the proposed measure to differentiate the strength of spatial attractiveness. Finally, if visualization is required, we can utilize combinations of primary colors (i.e., Red, Green, and Blue) to create single picture elements, or cells. For example, by assigning two features suitable color values of Red and Green, the composite *IC* network can be highlighted in Yellow that is the composite color of the Red and the Green in the real world. In this way, the co-location statistics can be represented by visual elements, instead of text files.
